# The importance of mucinous phenotype in colorectal carcinomas

**DOI:** 10.1186/1753-6561-6-S4-P15

**Published:** 2012-07-09

**Authors:** E Ozpinar, E Yanik, G Durmus, A Aydin, S Erdamar

**Affiliations:** 1 Department of Pathology, Istanbul University Cerrahpasa Medical Faculty, Istanbul, Turkey

## Introduction

Colorectal carcinomas (CRC) are one of the leading causes of cancer deaths, but their clinicopathological characteristics are not well documented in Turkish population. We retrospectively analyzed 535 resection specimens of patients diagnosed with primary CRC between the years of 2000-2007, to refine the pathological criteria for the routine analyses of colorectal cancer specimens. We also analysed that how mucin content can effect biological behavior in CRC cases.

## Methods

We retrospectively analysed the files of 535 consecutive patients diagnosed with primary CRC between the years of 2000-2007 and the specimens were obtained from archives of Cerrahpasa Medical Faculty, Pathology Department and were reevaluated. Clinicopathological features such as age; gender; size, location and histological grade of the tumor; presence and percentage of mucin in the tumor; lymphatic vessel invasion (LVI); blood vessel invasion (BVI); perineural invasion (PNI); total number of dissected lymph node (TLN) and lymph node metastases (LNM) and tumor invasion level (pT) were analyzed.

## Results

Mean age of patients was 64,2±10,5 (23-95) and female to male ratio was 0.8. Most common tumor locations were rectum and sigmoid colon in both females (28,4%; 22,3%) and males (29,1%; 24,1%), respectively. Only 3,5% of cases were younger than 40 years old. According to invasion level, 2,7% cases were T1 (n=14), 25% T2 (n=131); 36% T3 (n=189); 36,4% T4 (n=191). Invasion level was significantly correlated with tumor necrosis (p=0,000), LVI (p=0,000), BVI (p=0,042), PNI (p=0,000), LN metastases (p=0,005). Mean value of TLN was 16,1 (0-68) and LNM was 2,36 (0-56). 43% of all cases had lymph node metastases. Mean number of LNM was associated with higher invasion levels; 1,70 for T2, 1,89 for T3 and 2,01 for T4. Increased mucin secretion was correlated with increased level of tumor invasion (p=0,037); 29% for T2, 44% for T3 and 38% for T4. Tumors located in the ascending and transverse colon had no mucinous component. Tumors located more distally had higher extracellular mucin secretion.

## Conclusions

Our results indicate that clinicopathological characteristics of our series are similar to the other studies. The presence and degree of mucin secretion is related with the prognosis and therefore should be included in routine colorectal cancer pathology reports.

